# Contract-Optimization Approach (COA): A New Approach for Optimizing Service Caching, Computation Offloading, and Resource Allocation in Mobile Edge Computing Network

**DOI:** 10.3390/s23104806

**Published:** 2023-05-16

**Authors:** Zhiyao Sun, Guifen Chen

**Affiliations:** School of Electronic and Information Engineering, Changchun University of Science and Technology, Changchun 130000, China

**Keywords:** contract theory, resource allocation, computation offloading, service caching, heterogeneous networks, mobile edge computing

## Abstract

An optimal method for resource allocation based on contract theory is proposed to improve energy utilization. In heterogeneous networks (HetNets), distributed heterogeneous network architectures are designed to balance different computing capacities, and MEC server gains are designed based on the amount of allocated computing tasks. An optimal function based on contract theory is developed to optimize the revenue gain of MEC servers while considering constraints such as service caching, computation offloading, and the number of resources allocated. As the objective function is a complex problem, it is solved utilizing equivalent transformations and variations of the reduced constraints. A greedy algorithm is applied to solve the optimal function. A comparative experiment on resource allocation is conducted, and energy utilization parameters are calculated to compare the effectiveness of the proposed algorithm and the main algorithm. The results show that the proposed incentive mechanism has a significant advantage in improving the utility of the MEC server.

## 1. Introduction

In recent years, the rapid development of the Internet of Things (IoT) and artificial intelligence (AI) has led to the emergence of a wide range of complex applications, including augmented reality (AR) navigation, autonomous driving, face recognition, autonomous control, and smart healthcare [1]. However, these applications, which are typically latency-sensitive and computationally intensive, place significant demands on smart devices, which are unable to match these requirements for high CPU power and battery life [2]. Mobile edge computing (MEC) has thus emerged as a new paradigm to move computing away from centralized cloud infrastructure and toward the logical edges of the network, where base stations (BSs) with MEC servers installed, known as MEC-enabled BSs, are able to help mobile terminals (MTs) overcome their resource shortage [1]. At the same time, mobile edge computing helps large-scale environmental monitoring, is of great help in the prevention and control of natural disasters, and effectively improves the response efficiency of emergency communication networks [3].

In the realm of mobile edge computing (MEC), much attention has been devoted to task offloading. However, an often-overlooked aspect is the possibility of caching input data on the base station (BS), which can be reused by task offloading activities, leading to reduced costs associated with input data uploading [4]. Although repetitive task offloading operations for a mobile terminal (MT) are rare in practice, it is worth noting that the tasks generated by the MT are never precisely identical and always have distinct parameters. Nevertheless, the service components, such as databases, libraries, and programs that support task processing, can be shared and reused across the tasks of the MT. To address this issue, we propose a potential solution called service caching (similar to [5]), which entails caching essential service components on BSs. By enabling service sharing among the tasks of an MT, service caching effectively minimizes the amount of data required for uploading during the task offloading process, unlike data caching. However, the storage capacity of a BS is limited, allowing only a small number of services to be cached. Therefore, to optimize the system’s overall utility, it is vital to determine which services can be cached on the BS. Recent research has focused on jointly designing service caching, computing offloading, and resource allocation in MEC [1,6,7,8].

In addition, the standard cellular network is limited by constrained spectrum resources and cannot simultaneously support numerous devices offloading their computational workloads to MEC servers when the number of MTs increases. To address this challenge, HetNets, an emerging technology that deploys small cells (such as picocells and femtocells) atop microcells, have been proposed to increase spectral efficiency and system throughput by allowing small cells to repurpose subchannel resources of microcells. The effective control of interference and resource allocation strategies are crucial in HetNets due to the presence of co-tier and cross-tier interference. Recently, HetNets resource allocation, computational offloading, and service caching have all undergone collaborative design work, frequently employing MEC to reduce system latency, increase energy efficiency, or improve forecast accuracy (usually prediction accuracy will use computational performance evaluation metrics, such as RMSE [2,9,10,11]).

According to intuition, HetNets with MEC can effectively migrate compute offloading with service caching thanks to well-designed offloading methods. However, in reality, MEC services are provided by operators, and MTs must pay for this service. MTs are self-interested and want to maximize their profit, which makes it unrealistic to expect them to slavishly follow the control instructions from MEC servers. For this reason, it is crucial to design an effective incentive mechanism. Existing incentive mechanisms (such as Stackelberg game in [12] and auction in [13]) are currently used to allocate either cache, computation resources, or communication resources. However, no one has yet designed an incentive mechanism for optimizing multiple resources at the same time. This paper is the first to use contract theory (a potent paradigm from economics [14]) to simultaneously optimize multiple resources. The interaction between a MEC server and MTs with different classes of service cache-based computation tasks is formulated into a contract problem. The formulated problem maximizes the utility of the MEC server by optimizing CPU cycle, transmission power, caching decision, and reward while ensuring the non-negative and maximum utility for each MT selecting the contract item that belongs to their own type. The non-convex objective function and complex and non-convex constraints in the contract problem make the contract problem difficult to solve. By applying variable transformation and constraint reduction techniques, we transform and simplify the original contract problem, ultimately resolving it with a greedy algorithm.

The following is a list of this paper’s main contributions:

- Taking into account the information asymmetry where the MEC service provider is unaware of the MT’s transmission power and preference for delay, we apply contract theory to devise an incentive mechanism for the MEC service provider. Specifically, the four-dimensional contract item (including CPU cycle, transmission power, caching decision, and reward) can maximize the MEC service providers’ revenue gains while satisfying feasible individual rationality (IR) and incentive compatibility (IC) constraints for each MT. It is noted that IR and IC constraints can ensure the non-negative and maximum utility for each MT when they select the contract items that belong to their own type.
- We design a greedy algorithm to slove the formulated complex and non-convex contract problem. Due to the non-convex objective function and complex and non-convex constraints in the contract problem, we transform and simplify our contract problem through the methods of variable transformation and constraint reduction, and finally solve it using a greedy algorithm.
- The numerical results show that the proposed incentive mechanism has great advantages in improving the utility of the MEC service provider compared to other baseline mechanisms. In addition, we verify the validity of the proposed incentive mechanism and the greedy algorithm.

The rest of this paper is structured as follows. The related work of optimization scheme of service caching, computation offloading, and resource allocation in HetNets with MEC is presented in Section 2. The system model and problem formulation are presented in Section 3. The problem formulation and solution is presented in Section 4. Section 5 shows performance evaluation. Section 6 concludes this paper.

## 2. Related Work

Mobile edge computing (MEC) is a promising paradigm for addressing resource limitations in various aspects [15]. To mitigate computation, communication, and storage resource shortages, a variety of approaches have been proposed, including computation offloading and content caching strategies [16,17,18,19].

In [20], a stochastic mixed-integer nonlinear programming approach is presented, which optimizes decisions related to task offloading, wireless resource allocation, and elastic computing resource scheduling jointly. Mobile applications produce a vast amount of data at high rates, which can strain the backhaul link and mobile core network. Edge caching is an effective solution for storing and caching data at the mobile edge [21]. Moreover, it can handle spikes in mobile data traffic and enhance system performance [22,23]. Coordinated allocation of processing resources and communication between mobile devices and MEC servers is crucial to optimize system performance in heterogeneous networks. While recent efforts have been made to jointly design compute offloading and caching in MEC systems [1,6,7,8,11], the issue of edge server service utility rationalization has been neglected. Therefore, further research is required to optimize offloading and caching in heterogeneous network computing systems.

Most existing studies on incentive mechanisms for mobile edge computing (MEC) aim to encourage all parties to participate in the task-offloading system. Some recent studies have employed game theory to address resource allocation issues in heterogeneous networks. For instance, Li et al. [24] use deep reinforcement learning and game theory to maximize the MEC server’s profit while optimizing the choice of the MEC server, the size of the offloaded data, and the cost of the MEC computing service to prevent end users from abusing the system’s computing resources. An evolutionary-based MEC offloading system was proposed by Xia et al. [25], who also created an online distributed optimization method based on game theory and perturbed Lyapunov optimization theory. This algorithm decides how to manage battery energy as well as offload heterogeneous tasks and allocate compute resources as needed. However, these approaches only examine the joint optimization of computational and communication resources in the MEC system, neglecting cache resources. To address this, Tefera et al. [26] offer a congestion-aware distributed computing, caching, and communication architecture for MEC networks, using a deep reinforcement learning-based adaptive scheduling method based on noncooperative game theory to maximize each smart end-user device’s utility. Yan et al. [27] propose a MEC service incentive mechanism to control task offloading behavior and optimize service caching decisions, using a two-stage incomplete information dynamic game model and a simple algorithm to jointly optimize service providers’ pricing and service caching. Tutuncuoglu et al. [12] address the issue of offloading caches and pricing edge computing applications in a dynamic setting, representing the issue as a Stackelberg game. They propose a Bayesian Gaussian process bandit algorithm to learn the ideal pricing for a cache placement and greedy heuristic based on Gaussian process approximation to compute the cache placement in the situation of inadequate information, leading to significant performance improvements with low overhead.

Although game theory has been effective in addressing resource allocation, it often neglects user privacy. To address this, some researchers have used auction techniques. Le et al. [13] proposed a random auction technique to optimize social utility by simulating bandwidth transactions between suppliers and tenants. However, this approach has limitations, as it cannot foresee user demand or server failure, and the improvement in social welfare is not evident. In [28], the authors propose a dual-auction architecture that enables heterogeneous MECs to perform job offloading across edges without the use of cloud-centric servers. This approach benefits both social welfare and computation efficiency, but it does not consider resource allocation issues for communication and caching. Zhou et al. [29] used reverse auction as an incentive mechanism to model incentive-driven D2D offloading and content caching processes with the goal of maximizing cost savings for content service providers (CSPs). They proposed a content caching method based on deep reinforcement learning (DRL) and standard Vickrey–Clarke–Groves (VCG)-based payment rules to effectively save overhead and improve offloading efficiency.

Although the aforementioned working mechanism takes into account user privacy concerns and reflects individual rationality and incentive compatibility of participants, the auction mechanism is conducted periodically, and frequent interactions determine that new transactions occur. Therefore, the incentive mechanism is unsuccessful. The supply chain subjects can further increase transaction efficiency by creating procedures in the pre-transaction period using contractarianism to avoid frequent interactions with users. This is because the supply chain subjects are made up of numerous service-providing operators and customers [30]. Therefore, in [31], the authors first provide a contractarianism-based incentive mechanism that enables MEC operators to encourage more temporary ECNs to join the MEC network before they take into account the difficulty of allocating computational resources between ECNs and CSSs. Since CSSs contain private information, the issue is represented as a Bayesian matching game with externalities. The iterative matching method suggested next has the potential to significantly raise the amount of societal welfare.

Another line of research related to our work is the allocation of resources to heterogeneous networks based on other game theories. A new distributed computation offloading scheme for heterogeneous MECs was proposed by Wu et al. [32]. One of the early approaches was to formulate the problem as an optimization problem, accounting for inter-user interference and dynamic allocation of computation and communication resources when mobile devices (MDs) access different mobile stations (MSs). To minimize the overall system delay and energy consumption, a DOT computational offloading algorithm was developed that uses the finite improvement property of an ordinal potential game. The offloading problem is then modeled as a potential game, specifically an ordinal potential game. Moreover, since the freshness of information is very important in MEC applications, Yang et al. [33] considered the age of information (AoI) jointly with resource allocation in their work. For the purpose of obtaining AoI metrics, the authors first created a system model that takes active probability into account. They then established an AoI-based channel access optimization problem and used the ordinary potential game (OPG) approach to solve it. Finally, they proposed a learning algorithm called distributed channel access policy determination (DCASD) to choose the channel access policy. In [34], a dynamic and decentralized resource allocation technique is proposed based on evolutionary game theory to handle job offloading among different users to multiple edge nodes and central clouds. Replicator dynamics are utilized to simulate resource competition among several consumers. In [35], the authors propose an improved Gale–Shapley algorithm based on matching game theory for the issues of large resource differences and multiple-user service quality requirements in heterogeneous cellular networks. In heterogeneous networks, this algorithm can successfully reduce user service latency and enhance system performance.

As shown in Table 1, we summarize the related work on optimizing network resources through incentive mechanisms. We highlight the optimization strategy in this paper that uses contract theory as an incentive mechanism approach to jointly optimize CPU cycles, transmission power, and cache decisions with latency as the optimization objective. This emphasizes the need for an effective incentive mechanism that can safeguard the private information of MTs in HetNets with MEC.

## 3. System Model

In this section, we first present MEC in a HetNets environment that offloads computational tasks, some of which are transferred to the MEC servers and others are processed at the end device. Following that, the utility of MTs and MEC servers are described in more detail.

**Figure 1:**
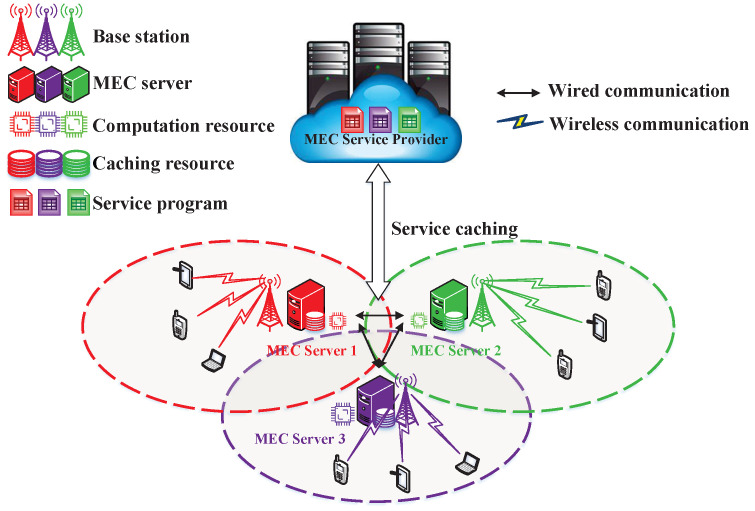
System illustration.

The system model shown in Figure 1 depicts a MEC system in HetNets that consists of an edge pool, denoted as $K=\left\{1,2,\cdots k,\cdots ,K\right\}$, and a number of MTs, denoted as $N=\left\{1,2,\cdots n,\cdots ,N\right\}$. The system includes *K* service programs that correspond to service-dependent tasks, and if a requested task’s associated service program has been cached at the edge pool or MTs, MTs can offload a portion of the computing tasks through wireless communication, such as cellular vehicle-to-everything (C-V2X), to the edge pool based on their trajectory and the location of the cached service program. The edge pool is composed of interconnected MEC servers that balance various computing and caching resources, but its limited computing and caching resources can only accommodate a small number of service programs being cached at the same time. Therefore, an AI-based management controller, or agent, is commonly deployed at the edge pool, which collects information from MTs and edge servers and makes decisions on service caching, task offloading, and resource allocation.

Next, we present in detail the utility of MEC servers and MT in the following scenario. In the scenario, a MEC service provider uses MEC servers to offer *K* classes of computational task to *N* MTs, with each class of computational tasks correlating to a separate service cache. At the same time, we think about the more real-world scenario when there is cross-terminal interference during task offloading. We define the number of MTs belonging to the class k∈K computational tasks as $N_{k}$, so we have $\sum_{k\in K}N_{k}=N$. Endpoints offload computational tasks to the MT in order to meet the task completion latency requirements due to the limited computing power of MTs. The offloaded computational tasks are required for service caching, and the edge server can choose to store them on the edge side or download them directly from the cloud center. We start by defining the MT as $n_{k}\in N_{k}$ for the class k∈K compute tasks. The task of MT $n_{k}\in N_{k}$ is denoted as $\left\{\theta_{k,n},D_{k},s_{k},\eta_{k},T_{k}^{\max}\right\}$, where $\theta_{k,n}$ denotes the sensitivity of the MT to the latency of the computation task of type *k*, $D_{k}$ is the data size corresponding to the computation task of type *k*, $s_{k}$ is the service cache size required by the computation task of type *k*, $\eta_{k}$ denotes the number of CPUs required for 1 bit of data for a computational task of type *k*, and $T_{k}^{\max}$ is the maximum tolerated latency for a computational task of type *k*. Here we assume that the maximum tolerable latency of offloading tasks of type *k* is the same. It is noted that MEC servers belong to MEC service providers, and in this paper we will use MEC service providers and MEC servers interchangeably. For ease of reference, Table 2 summarizes the key notations.

### 3.1. Utility of MT

The four phases that make up the MT $n_{k}$ offload task are as follows: the first step involves the MT uploading and transmitting the computation task to the edge server; the second involves the edge server obtaining the service cache either locally or from the cloud; the third involves the edge server executing the computation task; and the fourth involves the edge server transmitting the computation result to the MT. The fourth step takes very little time because the computation result is so little.

The upload transmission time of the first step is described as $\frac{D_{k}}{R_{k,n}}$, where $R_{k,n}$ is the MT’s wireless transmission rate, defined as

(1) $$R_{k,n}=B\log\left(1+\frac{d_{k,n}h_{k,n}^{2}}{\sigma^{2}+\sum_{b=1,b\neq n}^{b=N_{k}}d_{k,b}h_{k,b}^{2}+\sum_{a=1,a\neq k}^{K}\sum_{n=1}^{n=Na}d_{a,n}h_{a,n}^{2}}\right)$$

where σ is the Gaussian white noise, $d_{k,n}$ is the transmission power, and $h_{k,n}^{2}$ is the channel power gain between the edge server and the MT $n_{k}$. The second step’s time to acquire the service cache is $\frac{\alpha_{k}s_{k}}{R_{k}}$, where $\alpha_{k}=1$ denotes that the service caching is located in MTs, $\alpha_{k}=0$ denotes that it is located in the edge server. The third step’s computation time is $\frac{D_{k}\eta_{k}}{f_{k,n}}$, where $f_{k,n}$ is the edge server’s available CPU cycle. Based on the preceding instances, we define the MT satisfaction function $n_{k}$ as $\theta_{k,n}\left(T_{k}^{\max}-\frac{D_{k}}{R_{k,n}}-\frac{\alpha_{k}s_{k}}{r_{k}}-\frac{D_{k}\eta_{k}}{f_{k,n}}\right)$. The task is offloaded to the edge server by the MT $n_{k}$, which incurs a cost, defined as $p_{k,n}$. Therefore, the utility $u_{n,k}$ of the MT $n_{k}$ is defined as follows.

(2) $$\begin{array}{c} u_{k,n}=\theta_{k,n}\left(T_{k}^{\max}-\frac{D_{k}\eta_{k}}{f_{k,n}}-\frac{D_{k}}{R_{k,n}}-\frac{\alpha_{k}s_{k}}{r_{k}}\right)-p_{k,n} \end{array}$$

The channel gain between each MT, as well as the MT’s preference for the offloading task θ, are unknown to the edge server. For the same kind of offloading work, we first simplify the wireless transmission rate $R_{k,n}$ and account for the same power and gain for each MT. Hence, $R_{k}$ can be understood as follows.

(3) $$\begin{array}{c} R_{k}=B\log\left(1+\frac{d_{k}h_{k}^{2}}{\sigma^{2}+d_{k}h_{k}^{2}\left(N_{k}-1\right)+\sum_{a=1,a\neq k}^{K}N_{a}d_{a}h_{a}^{2}}\right) \end{array}$$

Following that, we investigate the contract design problem for the class *k* of the computing task.

**Definition** **1.** *There are $N_{k}$ MTs for the offload task type k on the edge server. The $N_{k}$ MTs can be classified into different latency sensitivity categories based on their type. The definitions are as follows $\Theta_{k}=\left\{\theta_{k,i}:1\leq i\leq I_{k}\right\}$. Because of this, there are $I_{k}$ classes of MTs in total that are within the edge server’s communication range. Each category’s probability distribution is $q_{k,i}$, and its corresponding number is $N_{k}q_{k,i}$, i.e., $\sum_{i\in I_{k}}N_{k}q_{k,i}=N_{k}$. Non-degenerate sequences of MTs are arranged according to kind.*

(4) $$\begin{array}{c} 0<\theta_{k,1}\leq \theta_{k,2}\leq \cdots \leq \theta_{k,I_{k}} \end{array}$$

A higher θ indicates that computational tasks should be offloaded to the edge server as soon as possible. In this case, we designate the contract for MT of type −i as $\left(f_{k,i},p_{k,i},d_{k},\alpha_{k}\right)$. The edge server will offer different contracts based on the θ of the MTs rather than providing the same contract to all MTs. MTs have the option to accept or reject any contract. MTs have the option to accept or reject any contract. If the MT rejects any contract, we assume that the MT signs a contract of (0,0).

To simplify the notation, we denote the MT within the edge server with offload task type *k* and time sensitivity type *i* as −(k,i). The utility of a MT of type −(k,i) is then redefined as follows.

(5) $$\begin{array}{cc} u_{k,i} & =\theta_{k,i}\left(T_{k}^{\max}-\frac{D_{k}\eta_{k}}{f_{k,i}}-\frac{D_{k}}{R_{k}}-\frac{\alpha_{k}s_{k}}{R_{k}}\right)-p_{k,i}\\ \; & =\theta_{k,i}G\left(f_{k,i}\right)-p_{k,i} \end{array}$$

where, $G\left(f_{k,i}\right)=T_{k}^{\max}-\frac{D_{k}\eta_{k}}{f_{k,i}}-\frac{D_{k}}{R_{k}}-\frac{\alpha_{k}s_{k}}{R_{k}}$.

### 3.2. Utility of MEC Servers

The expense to finish the computation task that the MT offloaded is borne by the MEC service provider. In order to coordinate with other MTs to lessen the effects of interference and to enable the MT to send data with $d_{k}$ power, the MEC service provider pays a unit cost of $c_{k,n}^{1}$ for the *k* class of offloaded computation tasks, i.e., the cost is $c_{k,n}^{1}d_{k}$. When the service cache is located in MTs, its cost is specified as $c_{k}^{2}\alpha_{k}r_{k}k$, where $c_{k}^{2}$ stands for the cost expenditure per unit transfer rate. This cost is paid by the MEC server. The cost of the service is specified as $c_{k}^{3}\left(1-\alpha_{k}\right)s_{k}$ when it is cached at the MEC server, where $c_{k}^{3}$ stands for the cost per unit of storage. The cost of computing to complete a task is denoted by the formula $c_{k}^{4}D_{k}\eta_{k}\kappa_{k,i}f_{k,i}^{2}$, where $c_{k}^{4}$ denotes the unit cost expenditure for computational energy consumption, $\eta_{k}$ is the arithmetic power needed by the MT $n_{k}$ to process a unit number of bits of data, and $\kappa_{n}$ is an effective switching capacitor. Thus, the utility obtained by the MEC server for the *k* class of offloaded computation tasks is as follows.

(6) $$\begin{array}{cc} U_{k}= & \sum_{i\in N_{k}}N_{k}q_{k,i}\left(p_{k,i}-c_{k,i}^{1}d_{k}-c_{k}^{2}\alpha_{k}d_{k}\right.\\ \; & \left.-c_{k}^{3}\left(1-\alpha_{k}\right)s_{k}-c_{k}^{4}D_{k}\eta_{k}\kappa_{k,i}f_{k,i}^{2}\right) \end{array}$$

As a result, the utility of MEC server is as follows for all types of computational jobs.

(7) $$\begin{array}{c} U=\sum_{k\in K}U_{k} \end{array}$$

## 4. Problem Formulation and Solution

### 4.1. Problem Formulation

In order to optimize MEC servers’ utilities, the MEC service provider provides incentives for multiple MTs to offload tasks to it. The MT is the agent that selects the contract item that best fits its kind, and the MEC service provider is the subject party who creates the contract. The contract for MEC servers is indicated as $\Phi_{k}=\left\{\left(\theta_{k,i},f_{k,i},p_{k,i},d_{k},\alpha_{k}\right),i\in I_{k}\right\}$, where $\left(f_{k,i},p_{k,i},d_{k},\alpha_{k}\right)$ is defined for MTs of type (k,i). For each MTs, they choose the contract that suits their type, satisfying both the individual rationality (IR) and incentive compatibility (IC) constraints.

While assuring a non-negative utility for each MT, the IR condition promotes MT involvement. The IR condition for MTs of type −(k,i) can be specifically expressed as

(8) $$\begin{array}{c} u_{k,i}\left(f_{k,i}p_{k,i},d_{k},\alpha_{k}\right)\geq 0,1\leq i\leq I_{k} \end{array}$$

The MT pays less than the gain from offloading work in order to encourage the MT to offload tasks to the edge server. The MT will decide not to offload the work and carry out the computation locally if for $u_{k,i}\left(f_{k,i},p_{k,i},d_{k},\alpha_{k}\right)<0$. The actual utility received by the MT of type −i is if the MT of type −i chooses the contract $\left(f_{k,j},p_{k,j},d_{k},\alpha_{k}\right)$ that is intended for the MT of type −j.

(9) $$\begin{array}{c} u_{k,i}\left(f_{k,j},p_{k,j},d_{k},\alpha_{k}\right)=\theta_{k,i}G\left(f_{k,j},d_{k},\alpha_{k}\right)-p_{k,j} \end{array}$$

As we previously established, our goal is to create a contract where MT of type −i chooses the $\left(f_{k,i},p_{k,i},d_{k},\alpha_{k}\right)$ contract over all other alternatives. In other words, MTs of type −i chooses the contract $\left(f_{k,i},p_{k,i},d_{k},\alpha_{k}\right)$ with the greatest utility. The following conditions must all be met for the contract to qualify as a self-revealing contract.

The IC condition ensures that each MT selects the contract that best meets its demands while still maximizing utility. The following equation can be used to determine the IC condition for type (m,i) MT.

(10) $$\begin{array}{c} u_{k,i}\left(f_{k,i},p_{k,i},d_{k},\alpha_{k}\right)\geq u_{k,i}\left(f_{k,i^{\prime }},p_{k,i^{\prime }},d_{k},\alpha_{k}\right),1\leq i,i^{\prime }\leq I_{k} \end{array}$$

The fundamental prerequisites necessary to guarantee contract incentive compatibility are the IC and IR limitations. Several additional requirements must be met in addition to the IC and IR restrictions.

**Theorem** **1.** *For k-th computation task, we have $f_{k,i}\geq f_{k,j}$ for each realizable contract $\left\{p_{k,i},f_{k,i}\right\}$ when and only when $\theta_{k,i}\geq \theta_{k,j}$. When, and only when, $\theta_{k,i}=\theta_{k,j}$, we have $f_{k,i}=f_{k,j}$.*

**Proof.**  Please refer to Appendix A.1 for proof.    □

**Definition** **2.** *Monotonicity: for k-th computation task, for any feasible contract {p,f}, the required computational resources f are as follows:*

(11) $$\begin{array}{c} 0\leq f_{k,1}<\cdots <f_{k,i}<\cdots <f_{I_{k}} \end{array}$$

*Higher latency-sensitive MTs, which call for more processing resources, are implied by monotonicity. We can obtain the following proposition by starting from the monotonicity’s nature.*

**Proposition** **1.** *The following conditions are intuitively satisfied by p as a strictly rising function of f.*

(12) $$\begin{array}{c} 0\leq p_{k,1}<\cdots <p_{k,i}<\cdots <p_{I_{k}} \end{array}$$

According to Proposition 1, incentive-compatible contracts cost more if they have a large computational capacity, and vice versa.

**Theorem** **2.** *For k-th computation task, each type of MT’s utility for each practicable contract {p,f} must be satisfied.*

(13) $$\begin{array}{c} 0\leq u_{k,1}<\cdots <u_{k,i}<\cdots <u_{k,I_{k}} \end{array}$$

**Proof.**  Please refer to Appendix A.2 for proof.    □

Now, we have $u_{k,i}>u_{k,j}$ when $\theta_{k,i}\geq \theta_{k,j}$. So, when $0<\theta_{k,1}<\theta_{k,2}<\cdots <\theta_{k,I_{k}}$, we have $0\leq u_{k,1}<\cdots <u_{k,i}<\cdots <u_{k,I_{k}}$.

As a result, MTs of higher types are more useful than MTs of lower types. Following is a simple conclusion that can be drawn from the IC requirement and the two lemmas we demonstrated. A lower obtained reward decreases the utility of the higher type MT if a higher type MT selects a contract intended for a lower type MT, even if the edge server allocates fewer computational resources for the tasks the higher type MT offloads. The cost outweighs the benefit if the low-type MT chooses a contract made for the high-type MT, since the gain from the high computational resources for the low-type MT’s computational activities cannot be balanced by its expense. The MT only achieves maximum utility when it selects the contract that most closely matches its preferences. We can thus promise that the contract is self-evident.

In the case of information asymmetry, the service provider, acting as the contract maker, must create the contract $\Phi_{k}$ for each MT in a way that maximizes its utility while fulfilling the IC and IR conditions. Consequently, the mathematical issue is framed as the following P1.

(14) $$\begin{array}{c} \begin{array}{c} \max_{p_{k,i,i}f_{k,i},d_{k},\alpha_{k}}U\\ \;s.t.\;C1:(\mathrm{IC}),\;\\ \;C2:(\mathrm{IR}),\;\\ \;C3:\;p_{k,i}\geq 0,1\leq i,i^{\prime }\leq I_{k},k\in K,\\ \;C4:\;f_{k,i}\geq 0,1\leq i,i^{\prime }\leq I_{k},k\in K,\\ \;C5:\;d_{k}\geq 0,k\in K,\\ \;C6:\;\alpha_{k}=\left\{0,1\right\},k\in K,\\ \;C7:\;\sum_{k\in K}\sum_{n_{k}\in N_{k}}N_{k}q_{k,i}f_{k,i}\leq F_{\max\;},\\ \;C8:\;\sum_{k\in K}\left(1-\alpha_{k}\right)s_{k}<S_{\max}. \end{array} \end{array}$$

where C7 indicates that the total amount of computing power used to carry out the computational activities offloaded by the MT cannot exceed the upper limit of the edge server’s computing power, and C8 indicates that the total amount of storage space utilized to store the service cache cannot exceed the upper limit of the MEC server’s storage space. The original optimization issue is not a concave problem and is difficult to solve, since the objective and constraints are not concave functions in P1.

### 4.2. Solution

We first simplify the original contract problem by using the following three theorems and then design an algorithm to solve the simplified contract problem.

We will use the following theorem for reducing IR constraints.

**Theorem** **3.** *$N_{k}$ IR constraints are reduced to one IR constraint of type 1.*

**Proof.**  From P1, we can see that a total of $N_{k}$ IR constraints are satisfied. However, we know from Definition 1 that $0<\theta_{k,1}\leq \theta_{k,2}\leq \cdots \leq \theta_{k,I_{k}}$. By using the IC constraint, we then have

(15) $$\begin{array}{c} \theta_{k,i}G\left(f_{k,i}\right)-p_{k,i}\geq \theta_{k,j}G\left(f_{k,1}\right)-p_{k,1}\geq \theta_{k,1}G\left(f_{k,1}\right)-p_{k,1} \end{array}$$

   □

Accordingly, satisfying the IR constraint of type 1 MT will automatically ensure the maintenance of the remaining IR constraints. Therefore, it is only necessary to maintain the first IR constraint while reducing the others.

We will use the following two theorems for reducing IC constraints.

**Theorem** **4.** *Downward incentive constraints (DICs) referring to IC constraints between types i and j where j∈1,⋯,i−1, are reduced to local downward incentive constraints (LDICs) referring to IC constraints between type i and type (i−1). The mathematical expression is as follows:*

(16) $$\begin{array}{c} \theta_{k,i}G\left(f_{k,i}\right)-p_{k,i}>\theta_{k,i}G\left(f_{k,j}\right)-p_{k,j},I_{k}\geq i>j\geq 1 \end{array}$$

**Proof.**  Please refer to Appendix A.3 for proof.    □

**Theorem** **5.** *Upward incentive constraints (UICs) referring to IC constraints between type i and type j where $j\in \left\{i+1,\cdots ,N_{k}\right\}$, are reduced to local upward incentive constraints (LUICs) referring to IC constraints between type i and type (i+1). The mathematical expression is as follows:*

(17) $$\begin{array}{c} \theta_{k,i}G\left(f_{k,i}\right)-p_{k,i}\geq \theta_{k,i}G\left(f_{k,j}\right)-p_{k,j},1\leq i<j\leq N_{k} \end{array}$$

**Proof.**  Please refer to Appendix A.4 for proof.    □

Based on Theorems 3, 4, and 8, P1 is reduced to P2 as follows.

(18) $$\begin{array}{c} \begin{array}{c} \max_{p_{k,i},f_{k,i},d_{k},\alpha_{k}}U\\ \;C2:\;\theta_{k,i}G\left(f_{k,i}\right)-p_{k,i}=\theta_{k,i}G\left(f_{k,i-1}\right)-p_{k,i-1}\\ \;C3:\;p_{k,i}\geq 0,1\leq i,i^{\prime }\leq I_{k},k\in K,\\ \;C4:\;f_{k,i}\geq 0,1\leq i,i^{\prime }\leq I_{k},k\in K,\\ \;C5:\;d_{k}\geq 0,k\in K,\\ \;C6:\;\alpha_{k}=\left\{0,1\right\},k\in K,\\ \;C7:\;\sum_{k\in K}\sum_{n_{k}\in N_{k}}N_{k}q_{k,i}f_{k,i}\leq F_{\max\;}\\ \;C8:\;\sum_{k\in K}\left(1-\alpha_{k}\right)s_{k}<S_{\max\;}. \end{array} \end{array}$$

Based on the C2 constraints in P2, we are able to derive

(19) $$\begin{array}{c} p_{k,i}=\sum_{z=1}^{i}\Delta_{z}+\theta_{k,1}G\left(f_{k,1}\right) \end{array}$$

where $\Delta_{1}=0$, $\Delta_{z}=\theta_{k,z}G\left(f_{k,z}\right)-\theta_{k,z}G\left(f_{k,z-1}\right),\forall z\in \left\{1,\ldots ,i\right\},\forall i\in \left\{1,\ldots ,I_{k}\right\}$.

By substituting the above equation into P2, we can obtain a transformed P3.

(20) $$\begin{array}{c} \begin{array}{c} \max_{f_{k,i},d_{k},\alpha_{k}}U=\sum_{k\in K}\sum_{i\in I_{k}}U_{k,i}\\ \;s.t.C1:\;f_{k,i}\geq 0,1\leq i,i^{\prime }\leq I_{k},k\in K,\\ \;C2:\;d_{k}\geq 0,k\in K,\\ \;C3:\;\alpha_{k}=\left\{0,1\right\},k\in K,\\ \;C4:\;\sum_{k\in K}\sum_{n_{k}\in N_{k}}N_{k}q_{k,i}f_{k,i}\leq F_{\max}\\ \;C5:\;\sum_{k\in K}\left(1-\alpha_{k}\right)s_{k}<S_{\max}. \end{array} \end{array}$$

where

$$\begin{array}{c} U_{k,i}=\theta_{k,i}G\left(f_{k,i}\right)\sum_{a=i}^{I_{k}}N_{k}q_{k,a}\\ -\theta_{k,i+1}G\left(f_{k,i}\right)\sum_{b=i+1}^{I_{k}}N_{k}q_{k,b}\\ -N_{k}q_{k,i}\left(c_{k,i}^{1}d_{k}+c_{k}^{2}\alpha_{k}d_{k}+c_{k}^{3}\left(1-\alpha_{k}\right)s_{k}\right)\\ -N_{k}q_{k,i}c_{k}^{4}D_{k}\eta_{k}\kappa_{k,i}f_{k,i}^{2},0<i<I_{k}; \end{array}$$

and

$$\begin{array}{c} U_{k,I_{k}}=\theta_{k,i}G\left(f_{k,i}\right)\\ -N_{k}q_{k,i}\left(c_{k,i}^{1}d_{k}+c_{k}^{2}\alpha_{k}d_{k}+c_{k}^{3}\left(1-\alpha_{k}\right)s_{k}\right)\\ -N_{k}q_{k,i}c_{k}^{4}D_{k}\eta_{k}\kappa_{k,i}f_{k,i}^{2},i=I_{k} \end{array}$$

The objective function in P3 can be expressed as $U=\sum_{k\in K}\sum_{i\in I_{k}}U_{k,i}$. Since the variables $f_{k,i},i\in I_{k},k\in K$ are independent of $d_{k},k\in K$ and $\alpha_{k},k\in K$, the optimization problem P3 is split into the following two subproblems. The subproblem P3.1 is the following:

(21) $$\begin{array}{c} \begin{array}{c} \max_{f_{k,i}}U_{1}=\sum_{k\in K}\sum_{i\in I_{k}}U_{1,k,i}\\ \;s.t.C1:\;f_{k,i}\geq 0,1\leq i,i^{\prime }\leq I_{k},k\in K,\\ \;C4:\;\sum_{k\in K}\sum_{n_{k}\in N_{k}}N_{k}q_{k,i}f_{k,i}\leq F_{\max} \end{array} \end{array}$$

where

$$\begin{array}{c} U_{1,k,i}=\left(\theta_{k,i}\sum_{a=i}^{I_{k}}N_{k}q_{k,a}-\theta_{k,i+1}\sum_{b=i+1}^{I_{k}}N_{k}q_{k,b}\right)\\ \left(T_{k}^{\max}-\frac{D_{k}\eta_{k}}{f_{k,i}}\right)-N_{k}q_{k,i}c_{k}^{4}D_{k}\eta_{k}\kappa_{k,i}f_{k,i}^{2},0<i<I_{k}, \end{array}$$

and

$$\begin{array}{c} U_{1,k,I}=\theta_{k,i}\left(T_{k}^{\max}-\frac{D_{k}\eta_{k}}{f_{k,i}}\right)-N_{k}q_{k,i}c_{k}^{4}D_{k}\eta_{k}\kappa_{k,i}f_{k,i}^{2},i=I_{k}. \end{array}$$

The subproblem P3.2 is the following:

(22) $$\begin{array}{c} \begin{array}{c} \max_{d_{k},\alpha_{k}}U_{2}=\sum_{k\in K}\sum_{i\in I_{k}}U_{2,k,i}\\ \;s.t.\;C2:\;d_{k}\geq 0,k\in K,\\ \;C3:\;\alpha_{k}=\left\{0,1\right\},k\in K,\\ \;C5:\;\sum_{k\in K}\left(1-\alpha_{k}\right)s_{k}<S_{\max}. \end{array} \end{array}$$

where

$$\begin{array}{c} U_{2,k,i}=\left(\theta_{k,i}\sum_{a=i}^{I_{k}}N_{k}q_{k,a}-\theta_{k,i+1}\sum_{b=i+1}^{I_{k}}N_{k}q_{k,b}\right)\\ \left(T_{k}^{\max}-\frac{D_{k}}{R_{k}}-\frac{\alpha_{k}s_{k}}{R_{k}}\right)\\ -N_{k}q_{k,i}\left(c_{k,i}^{1}d_{k}+c_{k}^{2}\alpha_{k}d_{k}+c_{k}^{3}\left(1-\alpha_{k}\right)s_{k}\right),0<i<I_{k}, \end{array}$$

and

$$\begin{array}{c} U_{2,k,I}=\theta_{k,i}\left(T_{k}^{\max}-\frac{D_{k}}{R_{k}}-\frac{\alpha_{k}s_{k}}{R_{k}}\right)-N_{k}q_{k,i}\left(c_{k,i}^{1}d_{k}+c_{k}^{2}\alpha_{k}d_{k}+c_{k}^{3}\left(1-\alpha_{k}\right)s_{k}\right),i=I_{k}. \end{array}$$

**Theorem** **6.** *The subproblem P3.1 is a convex problem.*

**Proof.**  It is important to note that $U_{1}$ is made up of concave inverse proportional and quadratic functions. These concave functions still have a concave shape when added together positively. Moreover, the constraints C1 and C4 are both affine sets, which means that it is a convex set. So the subproblem P3.1 is a convex problem.    □

Therefore, we can leverage standard convex optimization tools in [36] to solve it to obtain $f_{k,i}$.

**Theorem** **7.** *The subproblem P3.2 is a non-convex problem.*

**Proof.**  Since the subproblem P3.2 contains continuous variables (i.e., $d_{k},k\in K$) and integer variables $\alpha_{k},k\in K$ while $U_{2}$ in terms of $d_{k}$ is a non-convex function, this means that the subproblem P3.2 is a non-convex problem.    □

Since the subproblem P3.2 is a non-convex problem, finding the optimal solution usually requires exponential time complexity [36,37]. However, it is noted that $U_{2}$ in terms of $d_{k}$ is a concave function when $d_{j},\forall j\in K,j\neq k$ and π∈Π are both fixed. Here, Π is permutations of *K* service caches, one of Π is π. Thus, we have the following theorem

**Theorem** **8.** *The subproblem P3.2 is a concave problem in terms of $d_{k}$ when $d_{j},\forall j\in K,j\neq k$ and π are both fixed.*

**Proof.**  When $\alpha_{k},\forall k\in K$ and $d_{j},\forall j\in K,j\neq k$ are both fixed, $1+\frac{d_{k}h_{k}^{2}}{\sigma^{2}+d_{k}h_{k}^{2}\left(N_{k}-1\right)+\sum_{a=1,a\neq k}^{K}N_{a}d_{a}h_{a}^{2}}$ is a concave function. It is because its second order derivative is constantly less than zero when $d_{k}\geq 0$, which leads to $R_{k}=\log\left(1+\frac{d_{k}h_{k}^{2}}{\sigma^{2}+d_{k}h_{k}^{2}\left(N_{k}-1\right)+\sum_{a=1,a\neq k}^{K}N_{a}d_{a}h_{a}^{2}}\right)$ still being a concave function. Furthermore, we deduce that $-\frac{D_{k}}{R_{k}}$ is also a concave function. In addition, $-c_{k}^{2}\alpha_{k}d_{k}$ is also a concave function. The positive summation of all these concave functions, i.e., $U_{2}$, is still a concave function. Thus, $U_{2}$ in terms of $d_{k}$ is a concave function when $d_{j},\forall j\in K,j\neq k$ and π are both fixed. Moreover, the constraint set in terms of $d_{k}$ is a convex set. Finally, we complete the proof.    □

Theorem 8 motivates us to use a block coordinated descent (BCD) algorithm in [38] to find the optimal $d_{k},\forall k\in K$ when π is fixed. Further, a greedy algorithm is used to traverse each π to find the optimal π that maximizes the subproblem P3.2.

Thus, the algorithm for solving the subproblem P3.1 and the subproblem P3.2 is summarized as follows.

Initializing parameters and solving subproblem P3.1 using convex optimization tools have a time complexity of O($N_{k}^{3}$), where $N_{k}$ is the number of nodes in the network. Computing the permutations of *K* service caches takes O($2^{K}$) time. The while loop runs until the termination condition is met, i.e., $\epsilon <\epsilon_{\min}$, and we obtain $z^{max}$ and $z^{max}$ representing the number of iterations after the while loop in the ending pseudocode. After the above analysis, the final time complexity of Algorithm 1 is $O(z^{max}K2^{K})$.
**Algorithm 1** Finding the optimal contract items1:Initialize parameters2:Use standard convex optimization tools in [36] to solve subproblem P3.1 to obtain $f_{k,i}^{*},k\in K,i\in I_{k}$ and $U_{1}^{*}$.3:Compute the permutations of *K* service caches (denoted as Π)4:**for** Each π∈Π **do**5:    Initialize z=1, $d^{\pi ,z}=\left\{d_{1}^{\pi ,z},\ldots ,d_{k}^{\pi ,z},\cdots ,d_{K}^{\pi ,z}\right\}$6:    **while** $\epsilon >\epsilon_{\min}$ **do**7:        **for** Each service cache k∈K **do**8:           Use a greedy algorithm to find the optimal transmit power $d_{k}^{\pi ,z}$ when each transmit power $d_{a}^{\pi ,z},\forall a\in K,a\neq k$ is fixed.9:        **end for**10:        Compute $U_{2}^{\pi ,z}$11:        Compute $\epsilon =\left|\right|U_{2}^{\pi ,z}-U_{2}^{\pi ,z-1}\left|\right|$12:        Update z=z+1, $d^{\pi ,z}$13:    **end while**14:**end for**15:Find $\pi^{*}$ which maximizes $U_{2}$ and obtain $d_{k}^{*},k\in K$16:Based $\pi^{*}$ (i.e., $\alpha_{k}^{*},k\in K$), $d_{k}^{*},k\in K$ and $f_{k,i}^{*},k\in K,i\in I_{k}$, we can calculate $p_{k,i}^{*}$ by (19). Moreover, monotonicity is met automatically when the type is uniformly distributed [14].17:Output: $\alpha_{k}^{*},d_{k}^{*},f_{k,i}^{*},p_{k,i}^{*},k\in K,i\in I_{k}$

## 5. Simulation Results

We conducted simulations in MATLAB to demonstrate the effectiveness of the proposed incentive mechanism. In the simulation, we consider a MEC server offers K=3 classes of service cache-based computation tasks to N=15 MTs. The number of MTs for each class is equal, i.e, $N_{1}=N_{2}=N_{3}=5$. For fixed class, we set that the number of MTs as equal to the number of MTs’ types of preference for service latency, i.e., $N_{k}=I_{k}=5$. We assume that each type of preference for service latency has an equal probability within the range of (0,20], i.e., $q_{k,i}=\frac{1}{I_{k}}=\frac{1}{N_{k}}$. Additional parameters are presented in Table 3, which are based on prior studies [39,40,41,42].

Furthermore, the numerical results indicate that the proposed mechanism outperforms other baseline incentive mechanisms and significantly enhances the utility of the MEC server. These baseline incentive mechanisms are contract-based incentive mechanism under symmetric information scenario (CS), Stackelberg game-based incentive mechanism (SG) [43], and linear pricing incentive mechanism (LP). CS considers the scenario where the MEC server knows the types of preference of each MT. SG considers that the MEC server sets different unit price for each type of MTs. The objective of each MT is to maximize its own utility, which is expressed as

(23) $$\begin{array}{c} U_{k,i}^{\mathrm{SG}}=\theta_{k,i}G\left(f_{k,i}\right)-\delta_{k,i}f_{k,i} \end{array}$$

where $\delta_{k,i}$ is the price per CPU cycle. The objective of the MEC server is to maximize its own utility, which is expressed as

(24) $$\begin{array}{c} U^{\mathrm{SG}}=\sum_{k\in K}U_{k}^{\mathrm{SG}} \end{array}$$

where

$$\begin{array}{cc} U_{k}^{\mathrm{SG}}= & \sum_{n_{k}\in N_{k}}N_{k}q_{k,i}\left(\delta_{k,i}f_{k,i}-c_{k,i}^{1}d_{k}-c_{k}^{2}\alpha_{k}r_{k}\right.\\ \; & \left.-c_{k}^{3}\left(1-\alpha_{k}\right)s_{k}-c_{k}^{4}D_{k}\eta_{k}\kappa_{k,i}f_{k,i}^{2}\right) \end{array}$$

Referring to [44], LP considers that the MEC server sets same unit price for each type of MTs.

### 5.1. Algorithm Effectiveness

**Figure 2:**
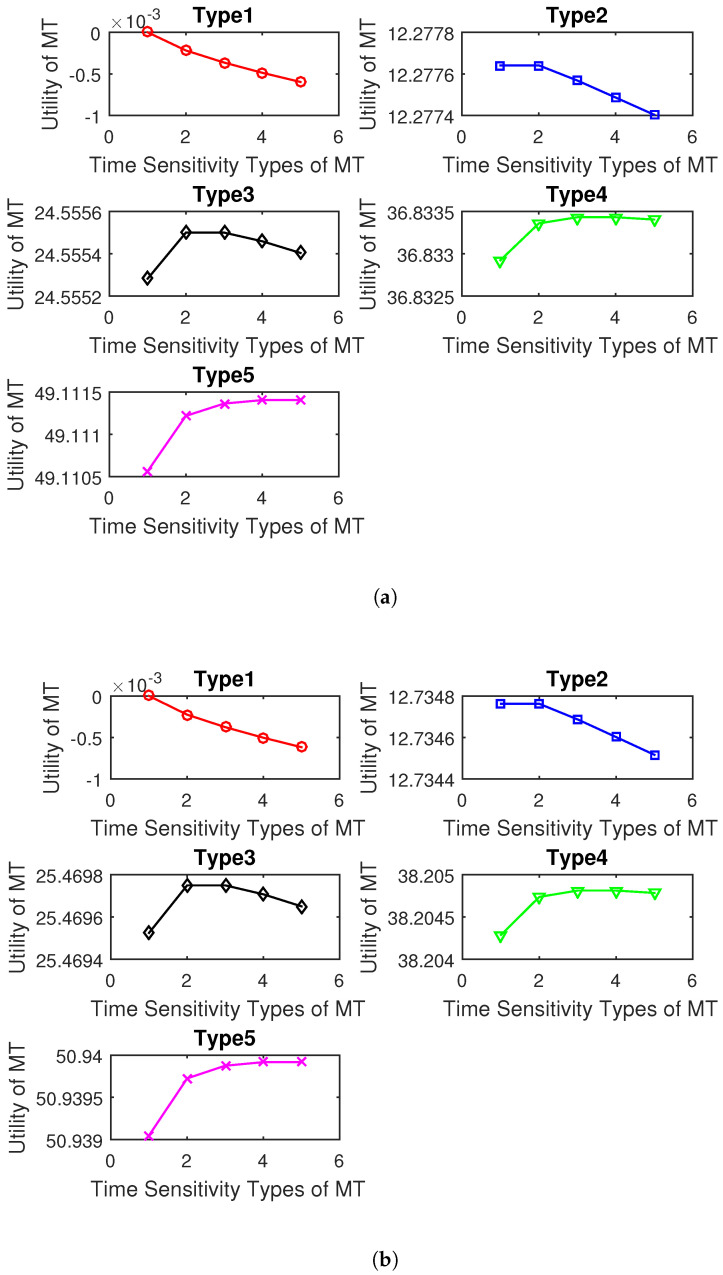

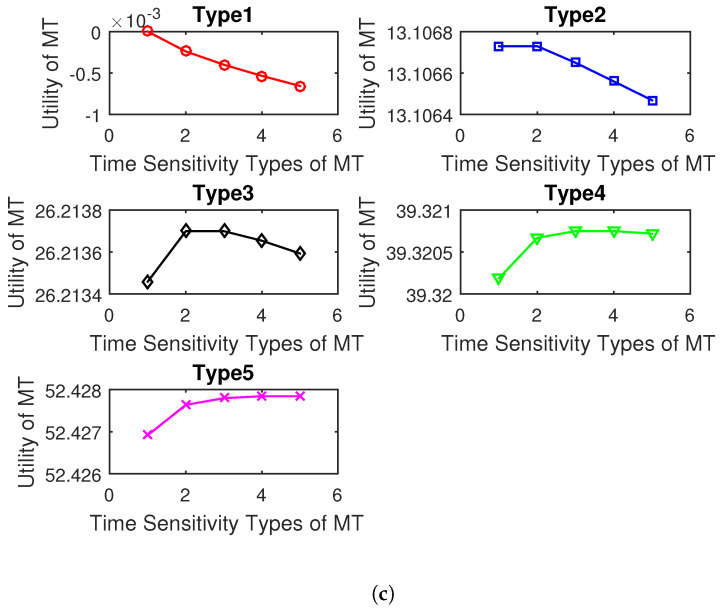
Types of MT versus utilities of MTs: (**a**) service caching type 1; (**b**) service caching type 2; (**c**) service caching type 3.

**Figure 3:**
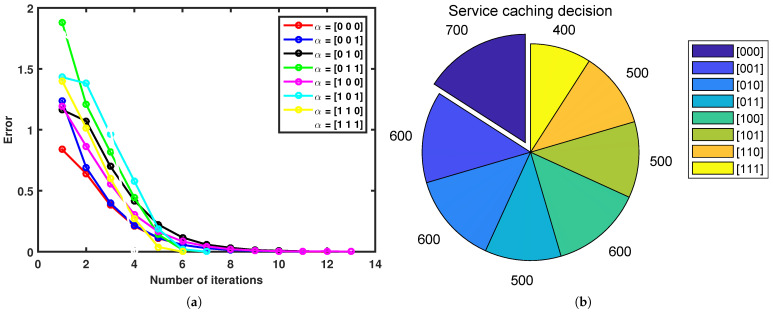
Algorithm convergence: (**a**) the predetermined error of the algorithm; (**b**) utility of various caching decisions.

In order to verify the effectiveness of the algorithm, we need to verify it in two steps. First, we verify the effectiveness of the optimal contract items as shown in Figure 2. Then, we verify the algorithm’s convergence as shown in Figure 3.

In Figure 2, we evaluate the IR and the IC conditions of our proposed CA scheme. Figure 2 shows the utilities of type-1, type-2, type-3, type-4, and type-5 MTs for different types of service caching when selecting all the contracts $\left(p_{k,i},f_{k,i}\right),k\in K,i\in I_{k}$ offered by the MEC server. For example, for service caching type 1, it can be seen that each MT can maximize its utility when selecting the contract that fits its own type, which means that the IC constraints are satisfied. Furthermore, each type of MT receives a positive utility value when selecting the contract that fits their type, which suggests that the IR constraints are satisfied. Therefore, by applying the proposed CA scheme, the MEC server can overcome the asymmetric information between the MEC server and the MTs by being aware of the types of MTs. Additionally, the utilities of higher types of MTs are larger than those of lower types of MTs, which verifies the result of Theorem 2.

Algorithm 1 converges to a predetermined error for P3.2 as the number of iterations increases, as demonstrated in Figure 3a, for various caching decisions. The MEC server can achieve various utilities depending on the caching decisions, as shown in Figure 3b. The maximum utility can be obtained by the MEC server when all service cache is stored on the MEC server, as demonstrated in Figure 3b.

### 5.2. Performance Comparison

In this paper, we compare our proposed contract-based incentive mechanism under asymmetric information (CA) to three other incentive mechanisms: CS, SG, and LP.

**Figure 4:**
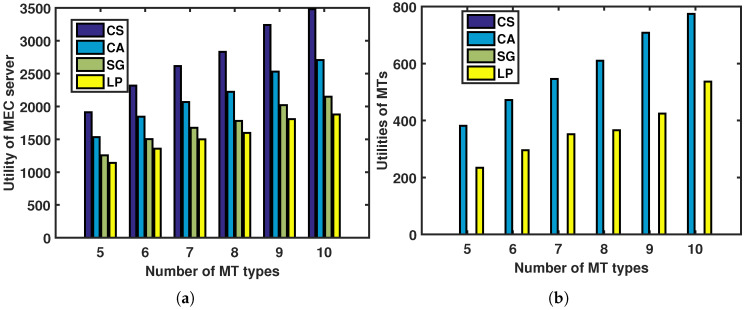
Utilities of MTs and MEC server versus number of MT types under different incentives: (**a**) utility of MEC server; (**b**) utilities of MTs.

Figure 4 depicts the relationship between the utilities of the MTs (and the MEC server) and the number of MT types under different incentive mechanisms. The utilities of the MTs and the MEC server increase with the number of MT types, as shown in Figure 4b and Figure 4a, respectively. Additionally, we analyze the utilities of the MTs and the MEC server under different incentive mechanisms when the number of MT types is fixed.

In Figure 4a, the CS approach achieves the best performance among the four approaches, serving as an upper bound. This is because the MEC server is fully aware of the different types of MTs and does its best to extract the maximum amount of revenue from them until their utilities are all zero. Additionally, the contract-based CS and CA methods outperform the SG approach in terms of the MEC server’s utility. The contract-based approaches aim to collect as much revenue from the MTs as possible while satisfying both the IR and IC constraints, leaving only a small share of revenue for the MTs. In contrast, the SG strategy aims to maximize the combined utility of the MEC server and the MTs, allowing for more revenue to be allocated to the MTs. Finally, the SG strategy outperforms the LP approach in terms of the MEC server’s utility, while the LP approach has the weakest performance among the four approaches. This is because the LP approach cannot adapt to changes in various offloaded jobs, which would worsen its performance. In Figure 4b, the CA approach provides better utilities for the MTs than the LP approach, while the utilities of the MTs are both zero under the SG and CS approaches. This is due to the same reasons mentioned in Figure 4a.

**Figure 5:**
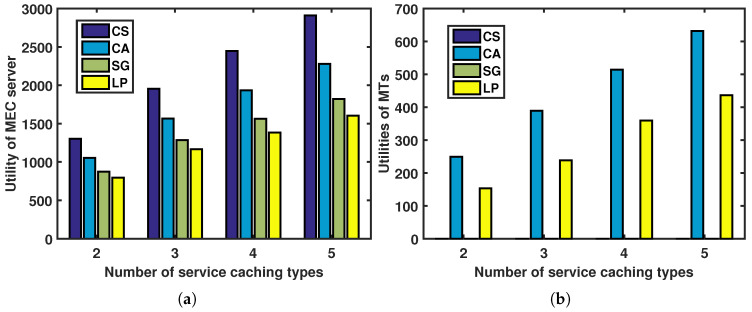
Utilities of MTs and MEC server versus number of service caching types under different incentives: (**a**) utility of MEC server; (**b**) utilities of MTs.

In Figure 5, the relationship between MTs’ utilities (and MEC server’s utility) and the number of service caching types is demonstrated under different incentives. The MEC server’s utility is shown to increase as the number of service caching types increases, as depicted in Figure 5a, and, similarly, the utilities of the MTs increase as well, as shown in Figure 5b.

**Figure 6:**
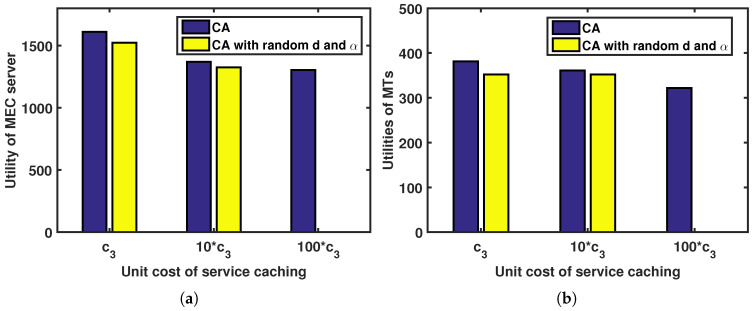
Utilities of MTs and MEC server versus unit cost of service caching under CA and CA with random strategies: (**a**) utility of MEC server; (**b**) utilities of MTs.

Figure 6 depicts the relationship between the utilities of the MTs and MEC server and the unit cost of service caching under the CA and CA with a random transmission power strategy *d* and caching strategy α. As shown in the figure, the MEC server’s utility decreases while the utilities of the MTs decrease along with the unit cost of service caching. Moreover, when the unit cost of service caching is fixed, the utilities of both the MTs and the MEC server are higher under the CA approach than under the CA with a random strategies approach.

**Figure 7:**
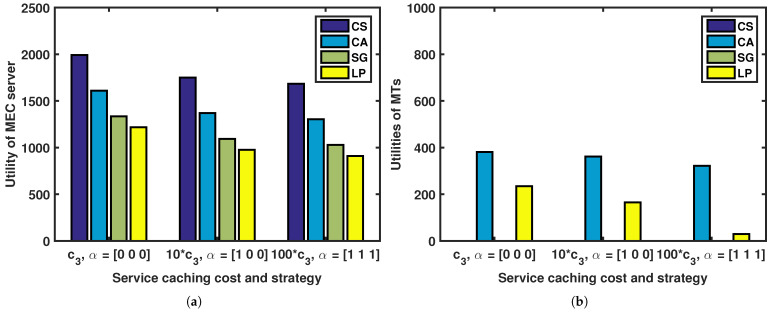
Effect of caching strategy and caching unit cost on utilities of MTs and utility of MEC server: (**a**) utility of MEC server; (**b**) utilities of MTs.

Figure 7 illustrates how the utilities of the MTs and MEC server are affected by caching strategy and caching unit cost. As shown, when caching costs are low, the MEC server tends to store all of its service caching on the edge side, represented by α=[000]. When caching costs are moderate, the MEC server tends to store part of its service caching on the edge side, represented by α=[100]. When caching costs are high, the MEC server tends not to store any of its service caching on the edge side, represented by α=[111].

**Figure 8:**
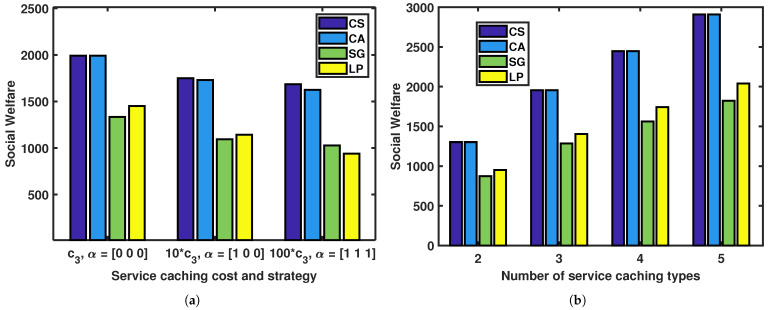
Effects of service caching costs and strategies and number of service caching types on social welfare: (**a**) service caching costs and strategies; (**b**) number of service caching types.

Figure 8 illustrates the effect of service caching costs and strategies and number of service caching types on social welfare. The social welfare is defined as

(25) $$\begin{array}{cc} U^{\mathrm{SW}} & =\sum_{k\in K}\sum_{i\in I_{k}}N_{k}q_{k,i}U_{k,i}+\sum_{k\in K}\sum_{i\in I_{k}}N_{k}q_{k,i}u_{k,i}\\ \; & =\sum_{k\in K}\sum_{i\in I_{k}}N_{k}q_{k,i}\left[\theta_{k,i}\left(T_{k}^{\max}-\frac{D_{k}}{R_{k}}-\frac{\alpha_{k}s_{k}}{R_{k}}\right)\right.\\ \; & \left.-\left(c_{k,i}^{1}d_{k}+c_{k}^{2}\alpha_{k}d_{k}+c_{k}^{3}\left(1-\alpha_{k}\right)s_{k}+c_{k}^{4}D_{k}\eta_{k}\kappa_{k,i}f_{k,i}^{2}\right)\right] \end{array}$$

where $U_{k,i}=p_{k,i}-c_{k,i}^{1}d_{k}-c_{k}^{2}\alpha_{k}d_{k}-c_{k}^{3}\left(1-\alpha_{k}\right)s_{k}-c_{k}^{4}D_{k}\eta_{k}\kappa_{k,i}f_{k,i}^{2}$. The social welfare in this context is measured by considering both the time gain and energy consumption. This is a more realistic approach as it takes into account the fact that users may prioritize either time or energy efficiency depending on their needs and preferences. The results suggest that the CS and CA methods can significantly improve social welfare compared to other methods, especially when the service caching costs and strategies are fixed. Moreover, increasing the number of service caching types can also lead to higher social benefits. Specifically, when the number of service caching types is the same, the CS and CA methods exhibit significantly higher social welfare than the other methods. It can be seen that the CS and CA methods can use less energy to produce the same time gain. Overall, these findings indicate the effectiveness of the CS and CA methods in enhancing social welfare and optimizing service caching in wireless networks.

## 6. Conclusions

In order to balance various computing capacities based on heterogeneous network environments, we construct a distributed heterogeneous network architecture in this study. Additionally, we create a method based on contract theory to optimize resource allocation, service caching, and compute offloading in order to maximize the revenue yield of MEC servers. We come to the conclusion that the contract problem is a complex problem with a non-convex objective function and complex non-convex constraints after a careful theoretical derivation. We use variable transformation and constraint reduction to transform and simplify the contract issue, then a greedy method is used to solve it. According to numerical data, the proposed incentive mechanism has a significant advantage over alternative baseline approaches in terms of increasing the utility of the MEC server.

## Figures and Tables

**Table 1:** Optimize network resources through incentive mechanisms.

Ref.	Optimization Strategies	Optimization Goals	Incentive Mechanism
[24]	Offloading data	Convergence time and stability	Deep reinforcement learning (DRL) and game theory
[28]	Utility of user	Social welfare and computation efficiency	Dual auction framework
[31]	Reward the MEC operator pays and CPU resource	Social welfare and computation efficiency	Contract theory and Bayesian matching game
[23]	Caching price and the number of contents stored on the edge caches	Quality of experience	Stackelberg game
[25]	CPU cycle and computation task offloading strategy and the unit task payment	Communication overhead and processing efficiency	Game theory and perturbed Lyapunov optimization
[26]	Variables for whether the tenant’s bid wins	Delay and energy consumption	Randomized auction mechanism
[27]	Service caching pricing	Prices and service caching decisions	Dynamic game of incomplete information
[12]	Offloading decision and transmission power	Computational overhead	Stackelberg game
In this paper	CPU cycle, transmission power, caching decision	Delay	Contract theory

**Table 2:** Key notations.

Symbol	Description
$K=\left\{1,2,\cdots k,\cdots ,K\right\}$	Set of edge nodes
$N=\left\{1,2,\cdots n,\cdots ,N\right\}$	Set of MTs
$N_{k}$	the number of MTs belonging to the class $\sum_{k\in K}$ computational tasks
$\theta_{k,n}$	The sensitivity of the MT to the latency of the computation task of type *k*
$D_{k}$	The data size corresponding to the computation task of type *k*
$s_{k}$	The service cache size required by the computation task of type *k*
$\eta_{k}$	The number of CPUs required for 1 bit of data for a computational task of type *k*
$T_{k}^{\max}$	The maximum tolerated latency for a computational task of type *k*
$R_{k,n}$	The terminal device’s wireless transmission rate
σ	The Gaussian white noise
$d_{k,n}$	The transmission power
$h_{k,n}^{2}$	The channel power gain between the edge server and the MT $n_{k}$
$\alpha_{k}$	Service cache location
$r_{k}$	The edge server’s transfer rate *m* for acquiring the service cache *k*
$f_{k,n}$	The edge server’s available processing power
$p_{k,n}$	The cost of tasks offloaded to edge servers
$u_{n,k}$	MT utility $n_{k}$
$\Theta_{k}=\left\{\theta_{k,i}:1\leq i\leq I_{k}\right\}$	The $N_{k}$ terminal devices can be classified into different latency sensitivity categories based on their type
$q_{k,i}$	Each category’s probability distribution
$c_{k,n}^{1}$	The unit cost the service provider pays for the computational tasks offloaded by the *k* class
$c_{k}^{2}$	The cost expenditure per unit transfer rate
$c_{k}^{3}$	The cost per unit of storage
$c_{k,n}^{4}$	The unit cost expenditure for computational energy consumption
$\beta_{k}$	The hotness level of the computational work delegated by the *k* type
γ	The file’s popularity
$\kappa_{n}$	The effective switching capacitor

**Table 3:** Parameter setting in the simulation.

Parameter	Setting
Effective switched capacitance	$\kappa =10^{-28}$
Number of CPU cycles executing one bit	η=β∈(0,10] cycles/bit
Maximum tolerance time	$T_{\max}\in \left(0,6\right]$ s
Size of service caching	s∈(0,81,920] bit
Maximum computing capacity	$F_{\max}=2\times 10^{10}$ cycles
Maximum storage capacity	$S_{\max}$ = 900,000 bit
Others	$c_{1}\in \left(0,1\right],c_{2}\in \left(0,5\right],c_{3}\in \left(0,10^{-6}\right]$

## Appendix A

### Appendix A.1

**Proof.** Using the IC constraint, we demonstrate this lemma. As a first step, we establish sufficiency: if $\theta_{k,i}\geq \theta_{k,j}$, then $f_{k,i}\geq f_{k,j}$. We start by defining $G\left(f_{k,i}\right)=T_{k}^{\max}-\frac{D_{k}}{f_{k,i}}-\frac{D_{k}}{R_{k}}-\frac{\alpha_{k}s_{k}}{r_{k}}$. IC constraint of MT according to type *i* and type *j*, where i≠j and $i,j\in I_{k}$, we have

(A1) $$\begin{array}{c} \theta_{k,i}G\left(f_{k,i}\right)-p_{k,i}\geq \theta_{k,i}G\left(f_{k,j}\right)-p_{k,j} \end{array}$$

and

(A2) $$\begin{array}{c} \theta_{k,j}G\left(f_{k,j}\right)-p_{k,j}\geq \theta_{k,j}G\left(f_{k,i}\right)-p_{k,i} \end{array}$$

The result of adding the two formulas above is $\theta_{k,i}G\left(f_{k,i}\right)+\theta_{k,j}G\left(f_{k,j}\right)\geq \theta_{k,i}G\left(f_{k,j}\right)+\theta_{k,j}G\left(f_{k,i}\right)$. We obtain $G\left(f_{k,i}\right)\left[\theta_{k,i}-\theta_{k,j}\right]\geq G\left(f_{k,j}\right)\left[\theta_{k,i}-\theta_{k,j}\right]$ by changing the formula’s terms. If we have $\theta_{k,i}\geq \theta_{k,j}$, then $\theta_{k,i}-\theta_{k,j}\geq 0$ must also exist. We obtain $G\left(f_{k,i}\right)\geq G\left(f_{k,j}\right)$ by dividing by the inequality’s two sides. We can infer that G(f) is a strictly growing function of *f* from the definition of G(·,·). We require $f_{k,i}\geq \cdot f_{k,j}$ to hold when $G\left(f_{k,i}\right)\geq G\left(f_{k,j}\right)$ does. Next, we prove the necessity: if $f_{k,i}\geq f_{k,j}$, then $\theta_{k,i}\geq \theta_{k,j}$. Similarly to the first case, using a similar procedure, we can obtain

(A3) $$\begin{array}{c} \theta_{k,i}\left[G\left(f_{k,i}\right)-G\left(f_{k,j}\right)\right]\geq \theta_{k,j}\left[G\left(f_{k,i}\right)-G\left(f_{k,j}\right)\right] \end{array}$$

Since $f_{k,i}\geq f_{k,j}$ and G(f) increase strictly with *f*, we must have $G\left(f_{k,i}\right)\geq G\left(f_{k,j}\right)$ and $G\left(f_{k,i}\right)-G\left(f_{k,j}\right)\geq 0$. Thus, by dividing the two sides of the inequality, we obtain $\theta_{k,i}\geq \theta_{k,j}$. As a result, we prove that there is $\theta_{k,i}\geq \theta_{k,j}$ when and only when $f_{k,i}\geq f_{k,j}$. Using the same procedure, we can easily prove that there is $\theta_{k,i}=\theta_{k,j}$ when and only when $f_{k,i}=f_{k,j}$. □

### Appendix A.2

**Proof.** When the constraints $\theta_{k,i}\geq \theta_{k,j}$ and $f_{k,i}\geq f_{k,j}$ are jointly applied, we can demonstrate from Definition 2 and Proposition 1 that we are aware that MTs requiring a delay-sensitive degree can gain more value by offloading the work. If the value is $\theta_{k,i}\geq \theta_{k,j}$, we have

(A4) $$\begin{array}{c} \begin{array}{c} u_{k,i}=\theta_{k,i}G\left(f_{k,i}\right)-p_{k,i}\geq \theta_{k,i}G\left(f_{k,j}\right)-p_{k,j}\left(\;\mathrm{IC}\;\right)↓\\ >\theta_{k,j}G\left(f_{k,j}\right)-p_{k,j}=u_{k,j} \end{array} \end{array}$$

□

### Appendix A.3

**Proof.** Since the number of MTs in our model is $N_{k}$, there exists a total of $N_{k}\left(N_{k}-1\right)$ IC constraints. Here, we focus on three different MT kinds that come after $\theta_{k,i-1}<\theta_{k,i}<\theta_{k,i+1}$. Then, we have the following two LDICs.

(A5) $$\begin{array}{c} \theta_{k,i+1}G\left(f_{k,i+1}\right)-p_{k,i+1}\geq \theta_{k,i+1}G\left(f_{k,i}\right)-p_{k,i} \end{array}$$

and

(A6) $$\begin{array}{c} \theta_{k,i}G\left(f_{k,i}\right)-p_{k,i}\geq \theta_{k,i}G\left(f_{k,i-1}\right)-p_{k,i-1} \end{array}$$

In Theorem 1, we prove that for any $\theta_{k,i}\geq \theta_{k,j}>0$, we have $f_{k,i}\geq f_{k,j}$, and the second inequality becomes

(A7) $$\begin{array}{cc} \; & \theta_{k,i+1}\left[G\left(f_{k,i}\right)-G\left(f_{k,i-1}\right)\right]\\ \; & \geq \theta_{k,i}\left[G\left(f_{k,i}\right)-G\left(f_{k,i-1}\right)\right]\\ \; & \geq p_{k,i}-p_{k,i-1} \end{array}$$

and

(A8) $$\begin{array}{cc} \; & \theta_{k,i+1}G\left(f_{k,i+1}\right)-p_{k,i+1}\\ \; & \geq \theta_{k,i+1}G\left(f_{k,i}\right)-p_{k,i}\\ \; & \geq \theta_{k,i+1}G\left(f_{k,i-1}\right)-p_{k,i-1} \end{array}$$

The above equation holds due to the fact that $\theta_{k,i+1}\left[G\left(f_{k,i}\right)-G\left(f_{k,i-1}\right)\right]\geq p_{k,i}-p_{k,i-1}$. Consequently, we have $\theta_{k,i+1}G\left(f_{k,i+1}\right)-p_{k,i+1}\geq \theta_{k,i+1}G\left(f_{k,i-1}\right)-p_{k,i-1}$. Thus, if LDIC holds for MTs of type −i, then the incentive constraint holds with respect to MTs of type −(i−1). This process can be extended from type i−1 down to type 1 to prove that all DICs hold.

(A9) $$\begin{array}{c} \begin{array}{c} \theta_{k,i+1}G\left(f_{k,i+1}\right)-p_{k,i+1}\geq \theta_{k,i+1}G\left(f_{k,i-1}\right)-p_{k,i-1}↓\\ \geq \cdots ↓\\ \geq \theta_{k,i+1}G\left(f_{k,1}\right)-p_{k,1},1\leq i\leq I_{k}↓ \end{array} \end{array}$$

□

### Appendix A.4

**Proof.** The following two LUICs result from the IC constraint.

(A10) $$\begin{array}{c} \theta_{k,i-1}G\left(f_{k,i-1}\right)-p_{k,i-1}\geq \theta_{k,i-1}G\left(f_{k,i}\right)-p_{k,i} \end{array}$$

and

(A11) $$\begin{array}{c} \theta_{k,i}G\left(f_{k,i}\right)-p_{k,i}\geq \theta_{k,i}G\left(f_{k,i+1}\right)-p_{k,i+1} \end{array}$$

In Theorem 2, we show that, for any value of $\theta_{k,i}\geq \theta_{k,j}>0$, we have the inequality $f_{k,i}\geq f_{k,j}$. This inequality may be deduced as

(A12) $$\begin{array}{cc} \; & p_{k,i+1}-p_{k,i}\geq \theta_{k,i}G\left(f_{k,i+1}\right)-\theta_{k,i}G\left(f_{k,i}\right)\\ \; & \geq \theta_{k,i-1}G\left(f_{k,i+1}\right)-\theta_{k,i-1}G\left(f_{k,i}\right) \end{array}$$

and

(A13) $$\begin{array}{cc} \; & \theta_{k,i-1}G\left(f_{k,i-1}\right)-p_{k,i-1}\geq \theta_{k,i-1}G\left(f_{k,i}\right)-p_{k,i}\\ \; & \geq \theta_{k,i-1}G\left(f_{k,i+1}\right)-p_{k,i+1} \end{array}$$

Therefore, we have

(A14) $$\begin{array}{c} \theta_{k,i-1}G\left(f_{k,i-1}\right)-p_{k,i-1}\geq \theta_{k,i-1}G\left(f_{k,i+1}\right)-p_{k,i+1} \end{array}$$

Therefore, all UICs are met if the incentive constraint on type −i consumers holds for MTs of type (i−1). All UICs assert that by extending this process upward from type (i+1) to $N_{k}$ MTs.

(A15) $$\begin{array}{c} \begin{array}{c} \theta_{k,i-1}G\left(f_{k,i-1}\right)-p_{k,i-1}\geq \theta_{k,i-1}G\left(f_{k,i+1}\right)-p_{k,i+1}\\ \geq \cdots \\ \geq \theta_{k,i-1}G\left(f_{k,N_{k}}\right)-p_{k,N_{k}},I_{k}\geq i>1 \end{array} \end{array}$$

□
